# Supplementary figures and images for: Host population dynamics influence Leptospira spp. transmission patterns among Rattus norvegicus in Boston, Massachusetts, US
Source: PLoS Negl Trop Dis. 2025 Apr 15;19(4):e0012966. doi: 10.1371/journal.pntd.0012966 (PMC12047771; doi:10.1371/journal.pntd.0012966)

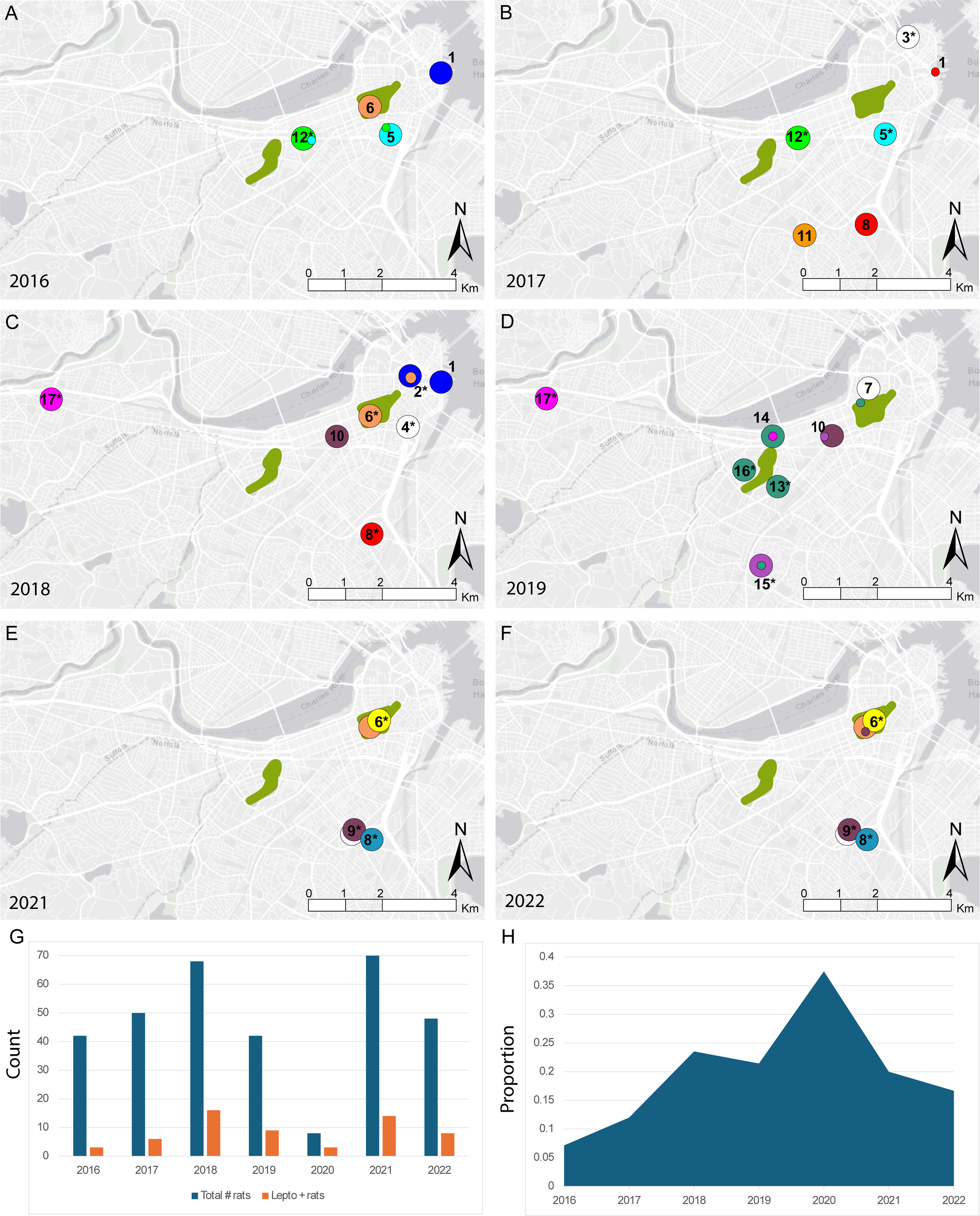

Supplement: S1 Fig — A-F) R. norvegicus collections (larger circles) displayed by sampling year and color coded according to genetic group (S1 Table). Inferred migrants are represented by smaller circles and lipL32 PCR positivity at 12 of 17 sites is indicated with asterisks. Major open spaces are indicated with green shading. G) Counts of total rats trapped (blue bars) and pathogenic Leptospira positive rats (orange bars) per year are displayed. H) The proportion of rats per year that were positive for pathogenic Leptospira spp. via lipL32 PCR. The maps in panels A-F were created using ArcGIS software by Esri. ArcGIS and Arc-Map are the intellectual property of Esri and are used herein under license. Copyright Esri. All rights reserved. For more information about Esri software, please visit www.esri.com. Basemap: Light Gray Canvas Base https://www.arcgis.com/home/item.html?id=8b3d38c0819547faa83f7b7aca80bd76. (TIF) [file pntd.0012966.s001.tif]

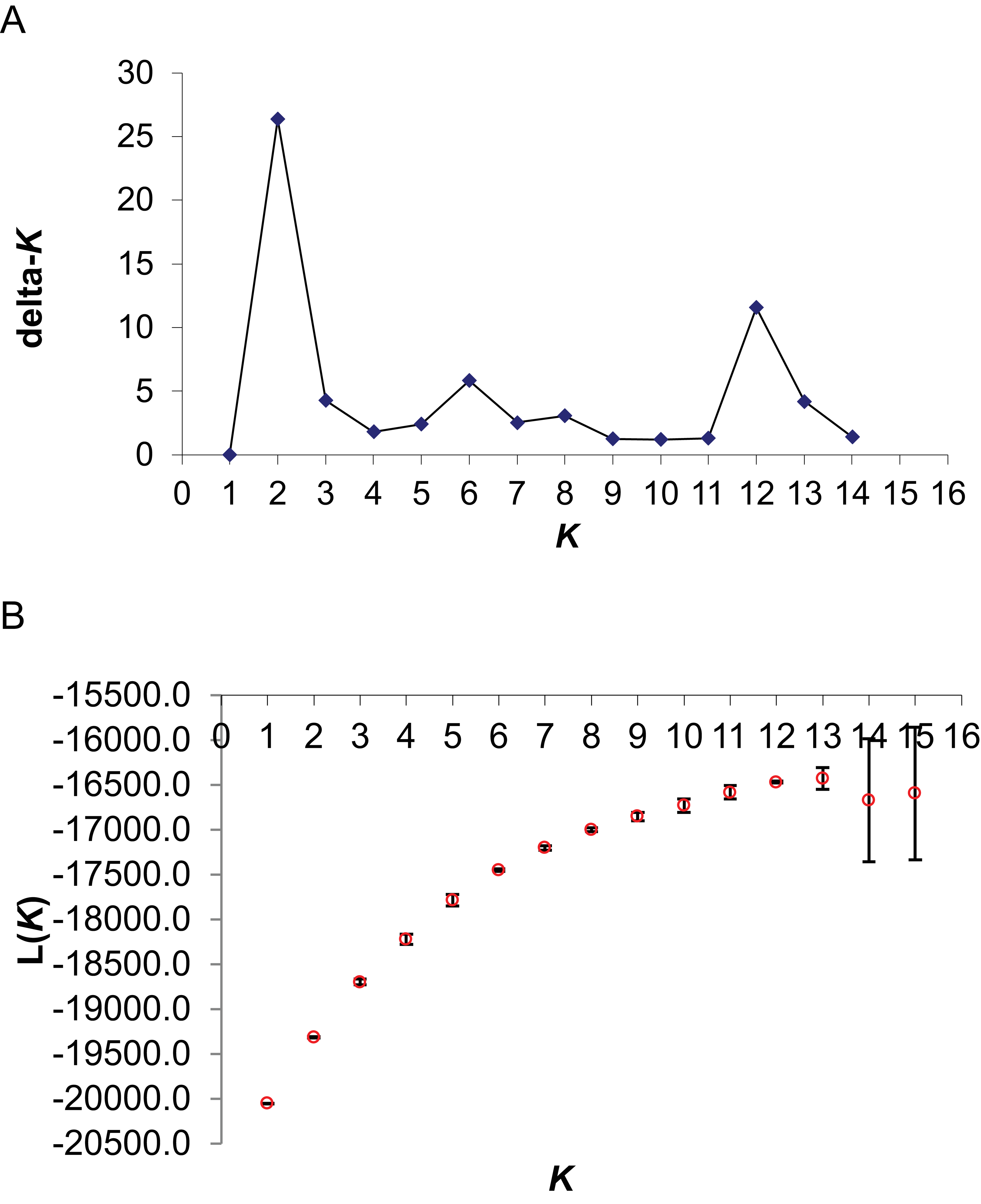

Supplement: S2 Fig — The hypothetical number of subpopulations (K) is plotted on the X-axis against A) delta-K, and B) The logarithm probability for each K [L(K)]. Ten independent runs in STRUCTURE were performed for each K value. (TIF) [file pntd.0012966.s002.tif]

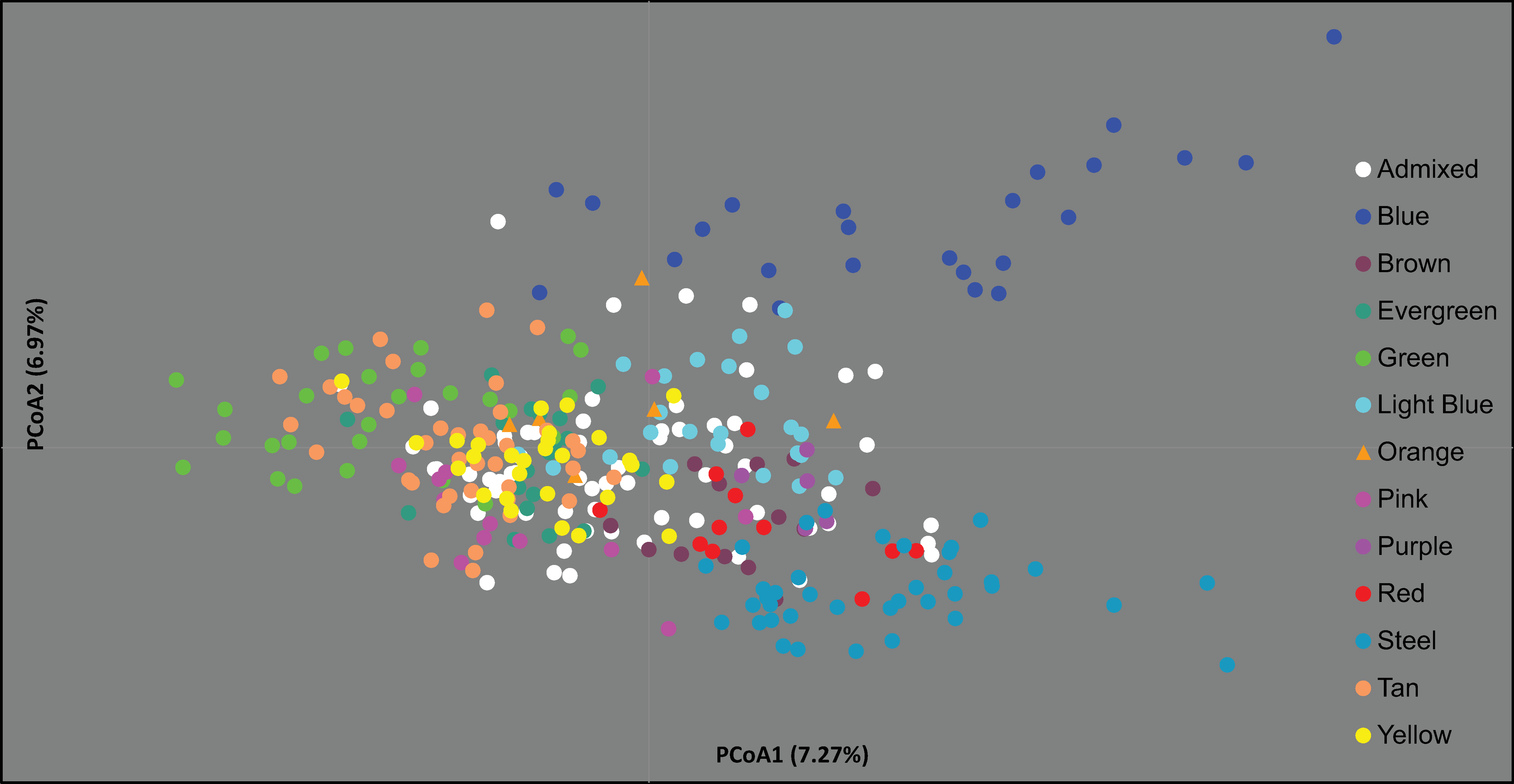

Supplement: S3 Fig — Principle Coordinates Analysis plot of standardized genetic distances illustrating genetic similarity among 311 Rattus norvegicus collected in Boston (2016–2022). Each rat is color coded according to its probability of membership (Q ≥ 0.75) to one of 12 genetic groups assigned using STRUCTURE (Fig 1 and S3 Table). The percentage of genetic variation explained by these two axes is indicated. (TIF) [file pntd.0012966.s003.tif]

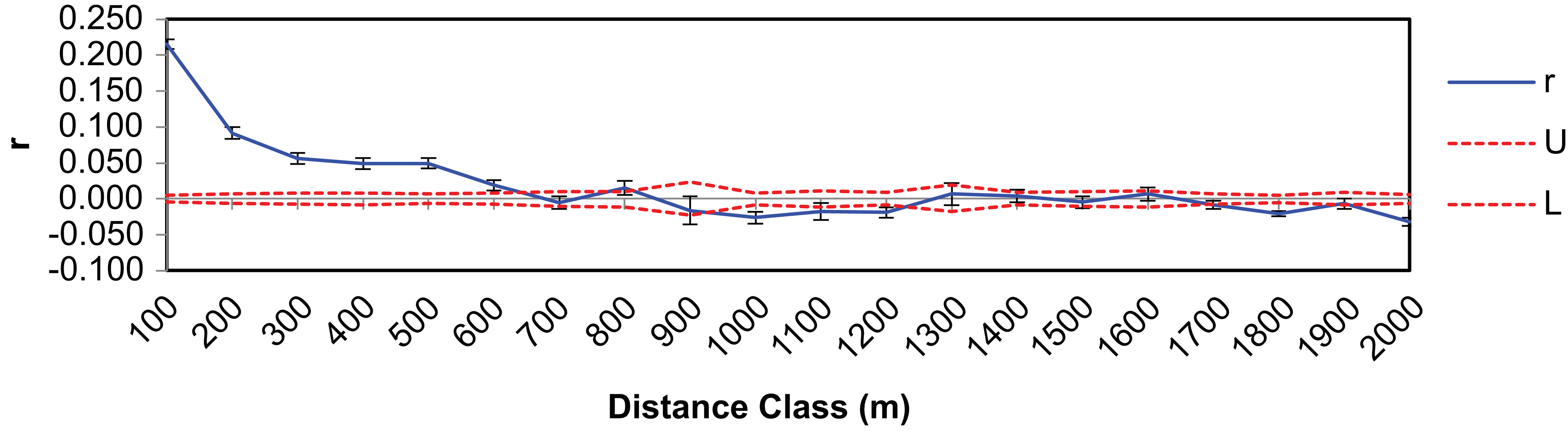

Supplement: S4 Fig — Spatial autocorrelation analysis graph assessing the correlation between genetic and geographic distance (Y-axis) using twenty even distance classes corresponding to 100 m intervals (X-axis). The blue line is the correlation value (r), and the red dashed lines are the 95% confidence intervals (U = upper and L = lower) about the null hypothesis of no spatial structure as determined by bootstrap resampling. Values of r above the upper 95% confidence interval are significant. (TIF) [file pntd.0012966.s004.tif]

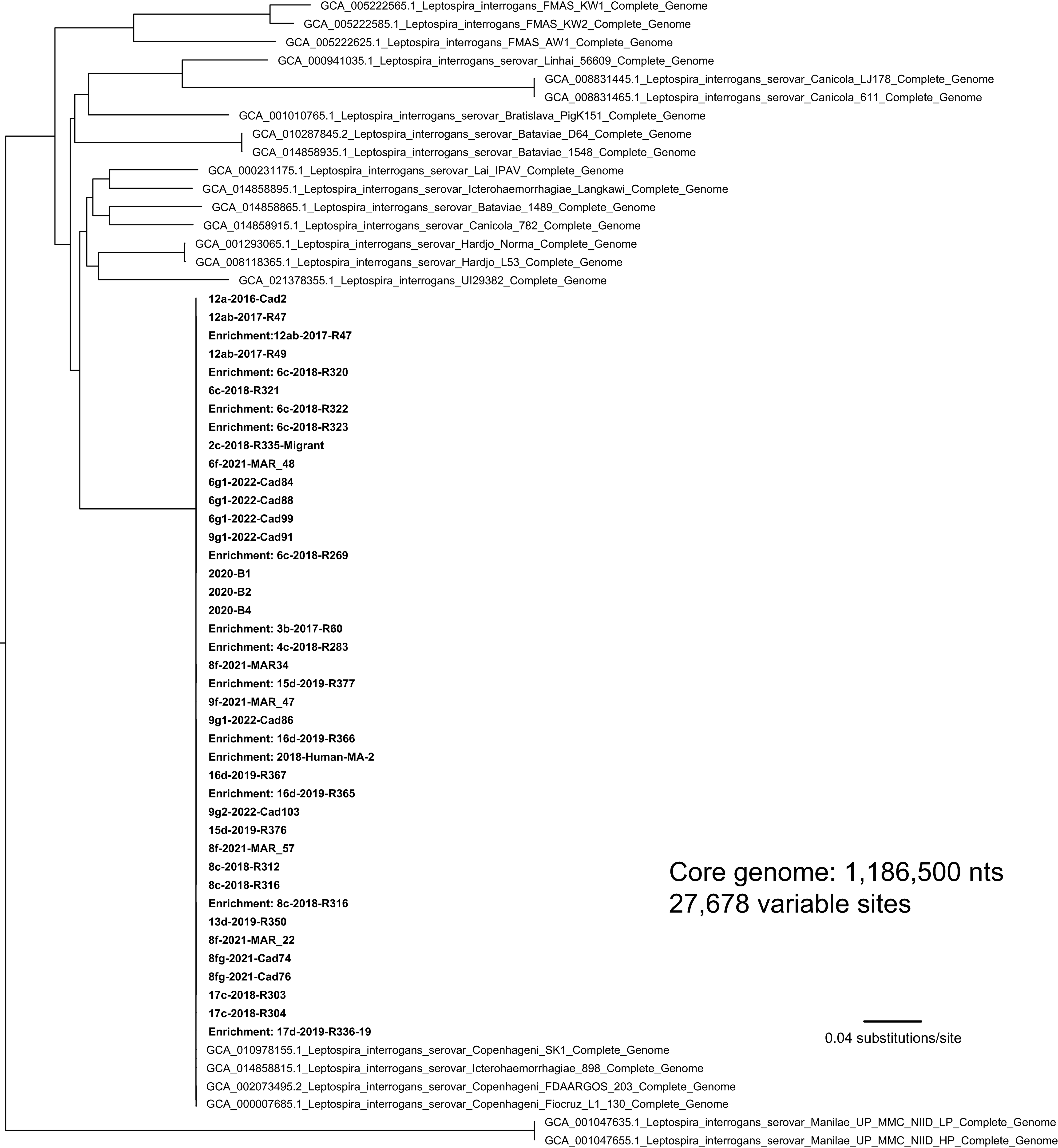

Supplement: S5 Fig — A maximum likelihood midpoint rooted phylogeny of 28 Leptospira interrogans serogroup Icterohaemorrhagiae isolate genomes and 13 enriched genomes from 40 rats and one human (bold text), together with 22 publicly available L. interrogans isolate genomes representing > 9 serovars (GenBank accession numbers are provided in the figure annotations), based upon a concatenated SNP alignment of 27,678 positions out of a core genome size of 1,186,500 nucleotides (nts). (TIF) [file pntd.0012966.s005.tif]

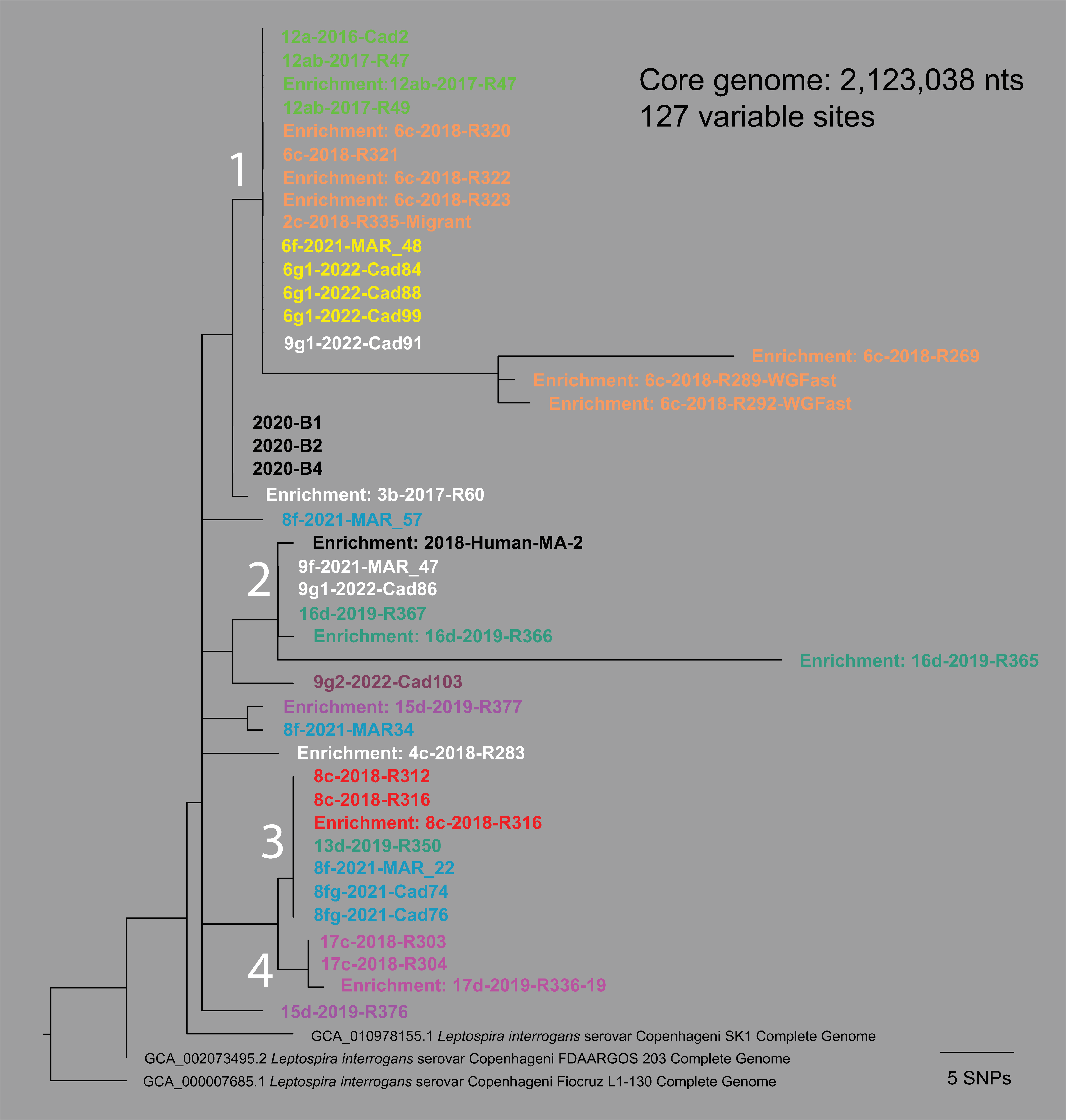

Supplement: S6 Fig — A maximum likelihood phylogeny of 28 Leptospira. interrogans serogroup Icterohaemorrhagiae isolate genomes from rats, together with two publicly available L. interrogans serogroup Icterohaemorrhagiae serovar Copenhageni complete genomes (GenBank accession# GCA_002073495.2 and GCA_010978155.1), and 13 enriched genomes (12 rats and 1 human), based upon a concatenated SNP alignment of 127 positions out of a core genome size of 2,123,028 nucleotides (nts). The phylogeny was rooted with reference genome L. interrogans serogroup Icterohaemorrhagiae serovar Copenhageni strain Fiocruz L1-130 (GenBank accession# GCA_000007685.1). The color of the genome ID corresponds to the rat genetic group in Fig 1; black text indicates that no genetic group was assigned. Two enriched samples (R289 and R292) had lower breadth and depth of coverage than the other enrichments (see S5 Table) and were inserted into the rat phylogeny based upon canonical SNPs with WG-FAST. (TIF) [file pntd.0012966.s006.tif]

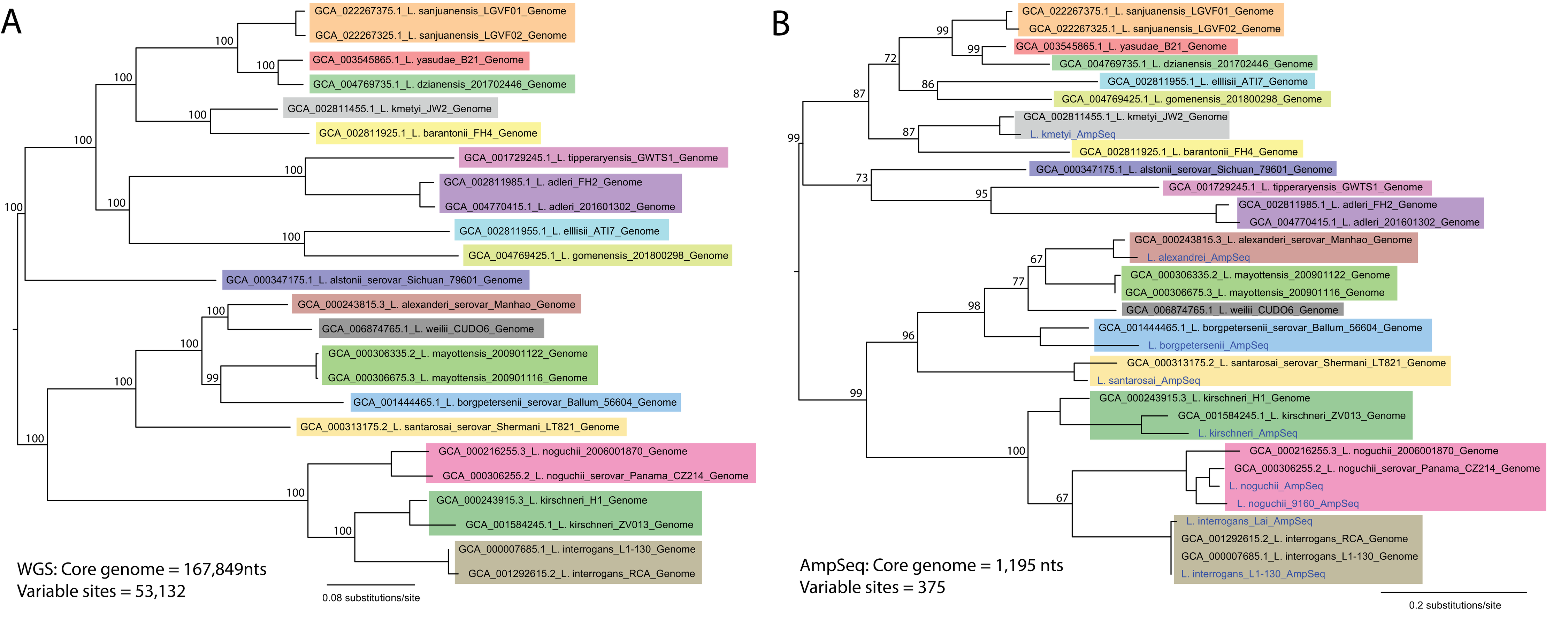

Supplement: S7 Fig — A) Whole genome maximum likelihood midpoint rooted phylogeny including 18 P1 clade pathogenic Leptospira spp. B) compared to an AmpSeq phylogeny that also includes AmpSeq data generated from nine Leptospira spp. isolate gDNAs (blue text). Species specific clades were maintained (color coded) and similar phylogenetic topologies were observed despite a large reduction in the core-genome of the AmpSeq tree (1,195 nts) compared to the whole genome tree (167,849 nts). Bootstrap values are indicated at specific nodes. The nine AmpSeq Leptospira spp. isolate gDNAs grouped in their respective species clades. (TIF) [file pntd.0012966.s007.tif]

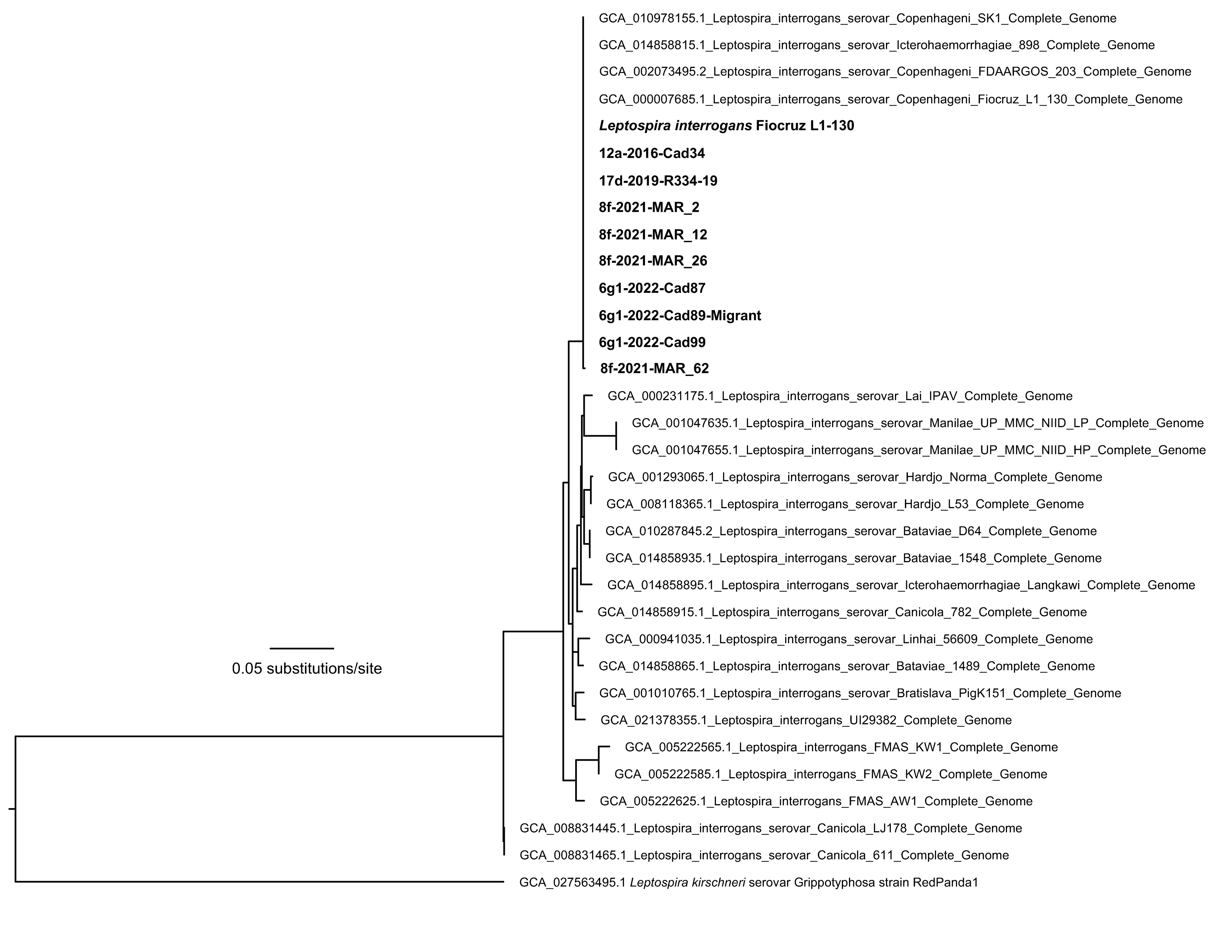

Supplement: S8 Fig — Maximum likelihood phylogeny based upon an alignment of 4,260 shared nucleotide positions using Leptospira spp. AmpSeq data generated from nine lipL32 qPCR positive Rattus norvegicus (bold text) along with 22 Leptospira interrogans isolate genomes (GenBank accession numbers are provided in the figure annotations). The phylogeny is rooted with Leptospira kirschneri serovar Grippotyphosa strain RedPanda1 (GenBank accession# GCA_027563495.1). (TIF) [file pntd.0012966.s008.tif]
