# Supplementary material for: Host population dynamics influence Leptospira spp. transmission patterns among Rattus norvegicus in Boston, Massachusetts, US
Source: PLoS Negl Trop Dis. 2025 Apr 15;19(4):e0012966. doi: 10.1371/journal.pntd.0012966 (PMC12047771; doi:10.1371/journal.pntd.0012966)
Supplement: Attachment — Submitted filename: Response to Reviewers_BostonRatLeptoMS.docx [file pntd.0012966.s020.docx]

Reviewer's Responses to Questions

Authors’ comment: We thank both reviewers for taking the time to review this article. We recognize that it is a long and comprehensive piece of work. As such, your comments are crucial to ensuring that we clearly communicate the findings from this study. Please note that the line references in our responses below correspond to the marked-up version of the revised manuscript.

-Are the objectives of the study clearly articulated with a clear testable hypothesis stated? -Is the study design appropriate to address the stated objectives? -Is the population clearly described and appropriate for the hypothesis being tested? -Is the sample size sufficient to ensure adequate power to address the hypothesis being tested? -Were correct statistical analysis used to support conclusions? -Are there concerns about ethical or regulatory requirements being met?

Reviewer #1: (No Response)

Reviewer #2: -Are the objectives of the study clearly articulated with a clear testable hypothesis stated?

Yes

-Is the study design appropriate to address the stated objectives?

Unclear. There is a tremendous amount of work presented in this manuscript, though I found the ultimate test of the stated hypothesis (does population structure of lepto match the structure of rat populations) more qualitative than quantitative. I would have expected a more quantitative analysis of spatial relationships of lepto isolates akin to what was performed for rats - if that had been performed, then I think a qualitative comparison of patterns would be acceptable inference. Instead, it appears that spatial structure of lepto isolates was based on the geographic placement of the rodents (or their identified cluster) from which they were isolated. Perhaps I missed a more explicit test of spatial relationships (?). At a minimum, spatial analyses of lepto isolates needs much more clarification than given.

Response: Thank you for this comment. Our hypothesis was not that the population structure of lepto matches the population structure of the rats, but rather that the population structure of the rats influences/drives Leptospira transmission among rats in Boston. We agree that we would need to analyze the spatial structure of the Leptospira as well, if that was the hypothesis we were testing. Rather, we are using genomic lineages (clades) of Leptospira interrogans found in Boston rats to understand the relatedness among the strains present in rats in Boston. Because these major clades represent different evolutionary pathways among these Leptospira interrogans strains, we can infer that they are “unique” strains and that these unique strains are associated with certain rat populations. All that to say, we agree that the analysis is qualitative and not quantitative. We have modified the text throughout to ensure that the hypothesis and goals of this study are clear.

-Is the population clearly described and appropriate for the hypothesis being tested?

Yes.

-Is the sample size sufficient to ensure adequate power to address the hypothesis being tested?

I believe so, though sampling effort is quite unequal and after following references, collected for different purposes based on the sampling time frame. Samples from 2021 - 2022 most closely match the stated hypothesis of the work. This should be discussed more thoroughly in the report.

Response: Thank you for this comment. Please see our response above

-Were correct statistical analysis used to support conclusions?

Yes

-Are there concerns about ethical or regulatory requirements being met?

No, though I fail to see the relevance of the pathogenic work performed in hamsters. I would perhaps recommend removing this section in its entirety.

Response: Thank you for this concern. The point of the hamster experiment was to validate the virulence of the isolate, and thus the role of the rodent (rat) in shedding a virulent organism that can cause acute disease e.g., in an incidental human host as reported to CDC.

-Does the analysis presented match the analysis plan? -Are the results clearly and completely presented? -Are the figures (Tables, Images) of sufficient quality for clarity?

Reviewer #1: (No Response)

Reviewer #2: -Does the analysis presented match the analysis plan?

Unclear. See comments above regarding methods.

Response: Thank you for this comment. Hopefully our description above and clarifying text in the manuscript addresses these concerns.

-Are the results clearly and completely presented?

Mostly. There are so many things reported that it is incredibly difficult to synthesis all of the work into a single, comprehensive report. See editorial comments below.

Response: We understand that there are a lot of methods, data, and analysis reported in this manuscript and appreciate your candid remarks regarding the difficulty in synthesizing this work. To simplify, we have identified unnecessary text throughout the manuscript and have deleted it wherever possible.

-Are the figures (Tables, Images) of sufficient quality for clarity?

I think too much information is packed into the figures. I would have liked to see more summary plots of sampling results (such as location/number/lepto positive samples per year). Anything to more clearly show a temporal/spatial trend that isn't tied to the complex genetic results that are presented. Could even be included as supporting material.

Response: This is an excellent idea. We have created a supplemental figure of summary plots, as suggested. Please see S1 Fig.

-Are the conclusions supported by the data presented? -Are the limitations of analysis clearly described? -Do the authors discuss how these data can be helpful to advance our understanding of the topic under study? -Is public health relevance addressed?

Reviewer #1: (No Response)

Reviewer #2: -Are the conclusions supported by the data presented?

No. I found the methods to identify dispersal inaccurate and I think the tie between spatial composition of lepto and rat populations could be more robust. At most, the data suggest relationships, but I am not convinced the relationships are as robust as stated.

Response: Thank you for expressing these concerns. We have significantly toned down our inferences of rat migrations driving Lepto transportation throughout the text because, as you state, in most cases these migrations are inferred based upon trapping locations. We have also attempted to clarify our hypothesis regarding the relationship between rat population dynamics and Leptospira transmission.

-Are the limitations of analysis clearly described?

No. Many other animals can transmit lepto, correct? Movement of pathogens by other reservoirs is not discussed.

Response: Yes, other animals can act as a reservoir for lepto and while that is true in general, it is not evident what other animal could possibly be acting as a reservoir host for this species/serogroup/serovar, L. interrogans serogroup Icterohaemorrhagiae [and likely] serovar Copenhageni, in such an environment i.e., We are not aware of any report that indicates dogs can act as a reservoir for this serogroup, nor foxes, possums, etc. And as the virulence of this pathogen is validated in the hamster, such hosts would likely suffer acute disease with this strain, rather than act as a reservoir.

-Do the authors discuss how these data can be helpful to advance our understanding of the topic under study?

Yes.

-Is public health relevance addressed?

Yes.

Editorial and Data Presentation Modifications? Use this section for editorial suggestions as well as relatively minor modifications of existing data that would enhance clarity. If the only modifications needed are minor and/or editorial, you may wish to recommend "Minor Revision" or "Accept".

Reviewer #1: (No Response)

Reviewer #2: See comments above regarding spatial analyses of lepto isolates.

In terms of the term "dispersal", what is stated as "observed" is merely "inferred". As best as I can understand, dispersal is identified by individuals from a certain location clustering with individuals from a different location at a set Q threshold.

Response: Thank you for this comment. We have changed “detected or observed” to “inferred” throughout the manuscript and removed much text regarding the influence of migrations on Leptospira transmission in this study system.

Related to dispersal, I think more caution needs to be used in especially in the context of lepto isolates. I infer from the paper that rats are driving all movement of lepto, which plausible, is complicated by the fact the other animals can be reservoirs of lepto. Movement by other reservoirs could wash out genetic signatures of the pathogens or provide a level of background noise that at least needs to be addressed in the discussion.

Response: See comment above addressing this concern. We have also added clarifying text in the discussion.

I think more consideration of K=2 should be discussed. Given sites 1 - 16 have much shorter spatial distances between them, just from looking at the map site 17 could be driving a lot of those spatial patterns. Did the authors look at the K=2 groupings

Response: Yes we did, see explanation in Results starting at line 225. The K=2 was not supported by Fst analysis and is often an artifact of STRUCTURE analyses because a K=1 is such a poor solution.

Summary and General Comments. Use this section to provide overall comments, discuss strengths/weaknesses of the study, novelty, significance, general execution and scholarship. You may also include additional comments for the author, including concerns about dual publication, research ethics, or publication ethics. If requesting major revision, please articulate the new experiments that are needed.

Reviewer #1: Study overview

This is an important, well written and a "very long" paper that describes the potential influence of rat population dispersal in urban environment on the transportation and diversity of Leptospira. The authors reported approximately 18% of Leptospira-positive samples from the tested samples, representing 12 of the 17 study sites. Despite evidence of high genetic structure, the study found a limited dispersal rate in the urban study sites. The authors' conclusion that the transportation and persistence of Leptospira in the environment are driven primarily by rat dispersal is questionable, given previous studies have indicated that adverse weather events such as flooding or even intense rainfall also significantly contribute to the same conclusion.

Response: Thank you for your careful review and these comments. The intention of this manuscript is not to infer that rats are driving ALL movement of lepto; rather this manuscript is one example of what can drive lepto, and the best example to study in this urban environment given the major role that rats play in similar environments throughout the world as a source of L. interrogans serogroup Icterohaemorrhagiae serovar Copenhageni, the reported leading species/serovar cause of acute infection in human patients globally. To clarify, we have modified the discussion to state that rat movement of Lepto in this urban setting is one possibility.

Additionally, the manuscript would benefit from addressing the following specific comments.

Comments to authors

*	Line 81: I would change "such as" to "mainly"

Response: This change has been made

*	Line 84: Consider changing "leptospirosis" to "the presence of Leptospira ..."

Response: This change has been made

*	Line 85: Change "movement" here to "transportation"

Response: This change has been made

*	Line 101: I will say "... other similar urban settings" here

Response: This change has been made

*	Lines 288 & 289: Could the author clarify how trapping efforts were accounted for here and also in the method, this should better reflect the population structure and dynamics than just relying on the number of rats captured at each site

Response: 6-10 traps were placed at each of the 64 trap locations. This is presented in the methods under section “Study location and rodent trapping”. Trapping spanned many years and was not uniformly implemented across all years and sites. Since the trapping effort (i.e. # traps placed during each trapping night) was not standardized and is not known for all trapping nights and sites, it is not calculated or used in the analysis.

*	Lines 412-414: It would be nice to state what the authors think might be responsible for this long-distance migration, especially even among adult females which is quite strange.

Response: We removed this text based upon reviewer 2’s comment that this migration was merely inferred rather than observed.

Additionally, please consider including how many of the migrant rats tested positive for Leptospira. This information could be valuable for understanding the role of rat dispersal in the spread/transportation of Leptospira pathogen

Response: Thank you for this comment. 2 of 11 inferred migrants were LipL32 positive. These data are presented in line 277.

*	Lines 415&416: Did the authors record any short-distance migration at these two sites, and for how long was sampling conducted at each site? This information is crucial given the distance among sites in this study and the fact that rats typically do not travel beyond 100m in urban environment. Without this information, it may somewhat represent a limitation of the study

Response: This section of the discussion has been removed to avoid reporting results that may be speculative. See additional comments/modification to “migrations” above

*	Lines 422 & 423: Consider to include how the rats were classified into juvenile and adult

Response: Juveniles were <140g. See line 848-849 in the methods

*	Lines 447 - 449: Could this also be associated with resource availability?

Response: Great comment. We have added this as a possible explanation on line 453

*	Lines 473-475: Please state how many of the positive rats were migrants

Response: The positivity rate was the same in inferred migrants, 18.2%. We have added these data on line 478.

*	Lines 562-566: The authors have not sufficiently supported their conclusion here. As mentioned earlier, it is essential to characterize how many of the migrant rats tested positive for Leptospira. Without this information, it is difficult to rule out alternative explanations, such as the transportation of L interrogans serogroup Icterohaemorrhagiae genotypes by flooding after rainfall which is common in literature

Response: Thank you for this comment. We have modified the text to reflect that migration is one possible explanation and have reported that 18.2% of inferred migrants were lepto positive

*	Line 645: group that ...?

Response: This sentence has been clarified

*	Lines 646 & 647: This statement requires cautions, in order not to over-blow the results

Response: This sentence has been removed

*	Lines 662 & 663: For how many nights were the traps placed at each site, and what were the criteria for placing specific number of traps at each site?

Response: We did not standardize the number of nights traps were placed at each site over the course of the study, but generally it was one night. However, the goal of trapping was to acquire rats not to estimate rat population abundance. Thus, we elected to proceed with opportunistic sampling to maximize our acquisition of biological samples. The criteria for the number of traps that were placed at each site were logistical based on placing them at least 15 meters apart in locations with evidence of rat activity, as stated in lines 671-673. Additional clarifying details have been added to this section of the methods in lines 671-686.

*	Line 673-675: Please include the procedure used for animal identification here

Response: A clarifying statement has been added to line 688-699. The animals were identified visually and the brown or Norway rat is the only species of rat that inhabits Boston.

*	Line 682 & 683: Add "pathogenic" before Leptospira

Response: This change has been made

*	Line 693: The authors should consider including a table with the sequences of the primers and probes used

Response: Thanks for this comment. These primers and probes are already well represented in the literature so we feel it would be redundant and unnecessary to include them here.

*	Line 694: Briefly describe the master mix reaction protocol with the concentrations of each reagent, the amount of DNA and the cycling program

Response: Again, this is an established PCR protocol that is already well represented in the literature so we feel it would be redundant and unnecessary to include here.

*	Lines 736-738: This way, on the average, how many samplings were conducted per year? Would it not be more appropriate to use term "sampling campaign" instead of the current term for clarity? The use of "collections" including in other parts of the manuscript can be confusing, as it may be interpreted as referring to the number of animals captured/collected. Please consider revising this terminology throughout the manuscript

Response: Thank you for noticing this. We have modified to “sampling campaign” where recommended.

Also, the term “collection” was meticulously defined in the methods and intended to be utilized throughout the manuscript to reflect that exact definition. We did find one instance where it was used incorrectly and have made that correction (see line 663). Thank you for your close attention to detail.

*	Line 757: Also, change "movement" to "transportation" here

Response: This change has been made as well as elsewhere in the manuscript

*	Lines 757-759: Considering the authors aims here, consider stating the frequency of trapping during the period of intensive sampling stated in Ln746 across the 3 sites. Also, briefly state the baiting and trapping protocol and for how long these lasted

Response: We have added more detail to the methods in lines 671-686 to address this.

*	Line 765: Where or were?

Response: Where is correct. We have added clarifying text

*	Lines 858-860: Considering that juvenile rats often wander more actively than matured rats ("Macdonald, D. W., Mathews, F., Berdoy, M., Singleton, G. H., & Leirs, H. (1999). Ecologically Based Rodent Management. The behaviour and ecology of Rattus norvegicus: from opportunism to kamikaze tendencies. Canberra: Australian Centre for International Agricultural Research, 49-80" and "<https://www.researchgate.net/publication/353608762_Using_Rhodamine_B_to_Assess_the_Movement_of_Small_Mammals_in_an_Urban_Slum>"), will the exclusion of juvenile rats not impact the result here, as their inclusion could provide insights into the overall population genetic diversity as oppose to just the genetic structure of only the breeding population?

Response: Thanks for this question/comment: The spatial structure analysis included all rats (adults and juveniles), Isolation by distance analysis also included juveniles and adults. The only analysis that excluded juveniles was inbreeding, which is appropriate. We have added clarifying text for each analysis.

Reviewer #2: This paper is essentially presenting three separate projects: 1) a longitudinal study of rats/lepto in boston, 2) a hyper-focused study of rat populations on either side of a suspected dispersal barrier, and 3) the development of new(er) technologies to isolate, amplify, and genotype lepto isolates. All three have merit on their own (though perhaps in different journals). Together, the main messages are lost, objectives get tied together when maybe they shouldn't be, and ultimately the stated hypothesis only matches a small subset of the sampling methodology (the 2021 - 2022 sampling). It reads as is that hyper-focused study was the ultimate driver of this work which is then supplemented by all of this other prior work that took place. I don't think there is anything wrong with that, but a lot of work more work needs to be put in to this manuscript to streamline what the ultimate objectives are. It could be the case that a lot of the methodology gets dumped into supporting information as it is ancillary to the objective of comparing population structure of lepto and rats.

Response: Thank you for these comments and we appreciate that this study is complex and bringing together several large, yet related datasets. We have made attempts to clarify our hypothesis throughout the manuscript and have removed convoluting text where possible. Because the methods are presented in the final section (after the discussion and conclusions) we do not perceive it necessary to move this text to supplementary materials as the reader can simply ignore this section, if desired.

Do you want your identity to be public for this peer review?

Reviewer #1: No

Reviewer #2: No

Figure Files:

While revising your submission, please upload your figure files to the Preflight Analysis and Conversion Engine (PACE) digital diagnostic tool, <https://pacev2.apexcovantage.com/>. PACE helps ensure that figures meet PLOS requirements. To use PACE, you must first register as a user. Then, login and navigate to the UPLOAD tab, where you will find detailed instructions on how to use the tool. If you encounter any issues or have any questions when using PACE, please email us at [figures@plos.org](mailto:figures@plos.org).

Data Requirements:

Please note that, as a condition of publication, PLOS' data policy requires that you make available all data used to draw the conclusions outlined in your manuscript. Data must be deposited in an appropriate repository, included within the body of the manuscript, or uploaded as supporting information. This includes all numerical values that were used to generate graphs, histograms etc.. For an example see here: <http://www.plosbiology.org/article/info%3Adoi%2F10.1371%2Fjournal.pbio.1001908#s5>.

Reproducibility:

To enhance the reproducibility of your results, we recommend that you deposit your laboratory protocols in protocols.io, where a protocol can be assigned its own identifier (DOI) such that it can be cited independently in the future. Additionally, PLOS ONE offers an option to publish peer-reviewed clinical study protocols. Read more information on sharing protocols at <https://plos.org/protocols?utm_medium=editorial-email&utm_source=authorletters&utm_campaign=protocols>

__________________________________________________

In compliance with data protection regulations, you may request that we remove your personal registration details at any time. (Use the following URL: <https://www.editorialmanager.com/pntd/login.asp?a=r>). Please contact the publication office if you have any questions.
