# Supplementary material for: Host population dynamics influence Leptospira spp. transmission patterns among Rattus norvegicus in Boston, Massachusetts, US
Source: PLoS Negl Trop Dis. 2025 Apr 15;19(4):e0012966. doi: 10.1371/journal.pntd.0012966 (PMC12047771; doi:10.1371/journal.pntd.0012966)
Supplement: Attachment — Submitted filename: Response to Reviewers_BostonRatLeptoMS_Feb25.docx [file pntd.0012966.s021.docx]

Author’s comment: Thank you for providing additional comments about areas of the manuscript that require further clarification.

• Kindly review and correct the manuscript throughout. Rats do not transport leptospirosis but rather the pathogen (Leptospira spp.), while the disease being transmitted is leptospirosis. I noticed that these two terms are occasionally used incorrectly in the manuscript.

Response: Thank you for pointing this out. The used of these terms has been addressed and standardized throughout the text.

• The phrase “6-10 trapping units were placed at each site...” is unclear. Did you mean that each site consisted of 6-10 trapping units, or that 6-10 traps were placed at each site? Additionally, the description “the trapping unit remained in place…” requires critical revision. Please provide a clear and concise explanation of what you mean by “trapping unit”, “trapping sites” and “trapping location” at the first mention to aid readers’ understanding.

Response: We have attempted to clarify these terms and their use in the text.

• Please cite a reference for this statement “Carcasses were visually identified as rats by the study team based on morphology and morphometry, and Rattus norvegicus is the only species of rat currently inhabiting Massachusetts”.

Response: This has been edited for clarity.

• Consider moving lines 736-742 under the study location and rodent trapping. You could further separate this sub-heading into “study area/description of study location” and “rodent trapping”. Then you can move the reasons for intensively sampling the selected sites up, so that the readers understand the rationale without having to wait to read several lines. It would be helpful to explain why the three areas were intensively sampled during the stated periods and not at another time.

Response: Thank you for this suggestion. Your suggestions for reorganization have been incorporated into the text.
